# Assessment of antibiotic prescribing in Latvian general practitioners

**DOI:** 10.1186/1471-2296-14-9

**Published:** 2013-01-12

**Authors:** Uga Dumpis, Elīna Dimiņa, Mārtiņš Akermanis, Edgars Tirāns, Sarmīte Veide

**Affiliations:** 1 Department of Infection Control, Pauls Stradins University Hospital, Pilsonu street 12, LV-1002, Riga, Latvia; 2 Association of Family Physicians, Latvia; 3 University of Latvia, Riga, Latvia

**Keywords:** Antibiotic use, General practitioners, Treatment of infection

## Abstract

**Background:**

Though general antibiotic consumption data is available, information on the actual patterns of prescribing antibiotics locally is difficult to obtain. An easy to use methodology was designed to assess ambulatory management of infections by Latvian general practitioners (GPs).

**Methods:**

GPs were asked to record data in a patient data collection form for every patient that received antibiotics. Study period – (7 days) one week in November, 2008. Data recorded included the following details: an antibiotic, the prescribed dose, dosing interval, route of administration combined with the demographic factors of the patient and clinical diagnosis based on a pre-defined list.

**Results:**

Two hundred forty eight forms out of the 600 (41%) were returned by post. Antibiotics were prescribed in 6.4% (1711/26803) of outpatient consultations. In total, 1763 antibiotics were prescribed during the study period. Ninety seven percent of the patients received monotherapy and only 47 (2.7%) patients were prescribed two antibiotics. The most commonly prescribed antibiotics were amoxicillin (33.9% of prescribed), amoxicillin/clavulanate (18,7%) and clarithromycin (7.6%). The most commonly treated indications were pharyngitis (29.8%), acute bronchitis (25.3%) and rhinosinusitis (10.2%). Pneumonia was mostly treated with amoxicillin/clavulanate (25,7%), amoxicillin (15.7%) and clarithromycin (19.3%).

**Conclusions:**

Methodology employed provided useful additional information on ambulatory practice of prescribing antibiotics and could be used in further assessment studies. Educational interventions should be focused on treatment of acute pharyngitis and bronchitis in children and unnecessary use of quinolones in adults for uncomplicated urinary tract infection.

## Background

The consumption of antibiotics is a major factor in the development of antibacterial resistance [1,2]. In addition, unnecessary use of antibiotics entails an increased risk of side effects [3-5] as well as additional costs [6].

Outpatient prescriptions account for the majority of their use [7,8]. Limited knowledge is available on the prescribing of antimicrobials in general population in countries where electronic prescription records are not available. Most studies investigating ambulatory consumption of antibiotics have been based on aggregated data from prescription databases or from wholesale figures. Such data usually does not contain information of indications for treatment [9]. Thus far the consumption of antibiotics and antimicrobial resistance rates of community acquired pathogens in Latvia have been among the lowest in the European countries [10-13]. Traditionally the general practitioners (GPs) have been the main antibiotic prescribers since they are at the centre of primary care system.

The aim of this study was to survey current outpatient antibiotic treatment practices for community-acquired infections in Latvian primary care by using a simple modified one-week prevalence protocol. Rather similar approach has been successfully used by investigators in the Scandinavian countries [7,9].

## Methods

In order to assess the antibiotics prescribed during the one study week at medical practice of GPs in Latvia, we used a modified one-week point prevalence approach with the protocol incorporating experience from earlier studies [4-8]. Study questionnaire was designed during the discussion with the GPs’ representatives on published protocols in relevance to the Latvian situation. The questionnaire was pilot tested on 20 GPs and several questions were abandoned due to the difficulties in understanding and additional workload. GPs were asked to record data for each patient that received antibiotics during one week in November, 2008. Six hundred questionnaires were handed out during the registration for a National conference.

Explanatory notes on the methodology were given during the presentation at the conference and written instructions were made available. The questionnaire contained questions regarding the antibiotics that were prescribed during the study week, the dose and dosing interval, the indications for use based on pre-defined diagnosis list and general demographic data (age, gender) including geographical region of GPs practices. The total number of all patients consulted during the study week was also collected. Participation was voluntary and did not involve financial incentives. The questionnaires were mailed back to the central office and data was entered in the system by data manager. ATC classification for antibiotics was used. The data was processed using EpiInfo 2005 and SPSS 16 software.

The study was approved by Pauls Stradins Clinical University Hospital Development Fund Ethical Committee as part of the National Research Programme. In accordance with this decision consent forms were not necessary since patient s’ and doctors information was not collected.

## Results

Six hundred questionnaires were distributed at the registration to general practitioners conference. Two hundred forty six (41%) questionnaires were returned by mail and submitted for further analysis. All regions of Latvia were represented in the pool of returned questionnaires with most of them (101) coming from capital city Riga. This was representative of the population size distribution within the country. Urban, semi-urban and rural practices were represented in the study sample.

During the study week, antibiotics were prescribed at 6.4% (1711/26803) of general practitioners’ consultations. Two hundred five antibiotics were prescribed during a home visit. The mean number of consultations was 106 per GP (range 12 – 240). The mean number of antibiotic prescriptions prescribed per GP was 7.2. Fifty six percent of patients were females.

The mean age of the patients who were prescribed an antibiotic was 31.1 (range < 1 to 97 years), 13% of all patients treated were > 60 years of age and the greatest number of the antibiotic prescriptions (24.1%) of all age groups were given to children younger than 10 years of age.

The mean duration of the antibiotic therapy prescribed was 6.9 (SD+/− 4.8) days, 41% and 33.7% of all prescriptions were for seven and five days, respectively and only in 1.8% of the patients were prescribed a three-day course.

Overall, a total of 33 different antibiotics were prescribed (Table 1). In children younger than 10 years, 21 different antibiotics were prescribed. Ninety seven percent of patients received monotherapy, and only 47 (2.8%) patients were prescribed two antibiotics. The most common combinations were amoxicillin and clarythromycin prescribed for respiratory tract infections (21/47) and clarithromycin and metronidazole prescribed for gastrointestinal (7/47) (apparently *Helicobacter pylori)* infections. For most of the patients were prescribed *per os*, but in 14 (0.8%) patients antibiotics were prescribed intramuscularly and in 20 (1.1%) intravenously. Antibiotics prescribed for parenteral use were ceftriaxone J01DD04 and gentamicin J01GB03.

**Table 1:** Prescribed antibiotics according to patients’ age

**Age group**	**<5**	**5-14**	**15-64**	**> = 65**	**Total**	DDD*
	**N(%)**	**N(%)**	**N(%)**	**N(%)**	**N(%)**	**DDD (%)**
Penicillins with extendedspectrum (J01CA)	113 (42,8)	119 (44,6)	329 (31,9)	48 (24,4)	609 (34,6)	649,8 (35,3)
Amoxicillin/clavulanate (J01CR)	37 (14,0)	42 (15,7)	220 (21,3)	30 (15,2)	329 (18,7)	416,6 (22,7)
Macrolides (J01FA)	38 (14,4)	31 (11,6)	154 (14,9)	24 (12,2)	247 (14,0)	270,7 (14,7)
Trimetroprim/sulfametoxazole (J01EE)	49 (18,6)	20 (7,5)	36 (3,5)	4 (2,0)	109 (6,2)	35 (1,9)
First generation cephalosporins (J01DB)	17 (6,4)	24 (9,0)	55 (5,3)	11 (5,6)	107 (6,1)	38,3 (2,1)
Quinolones (J01MA)	0	3 (1,1)	81 (7,8)	33 (15,7)	117 (6,5)	185,1 (10,1)
Tetracyclines (J01AA)	0	5 (1,9)	57 (5,5)	20 (10,2)	82 (4,7)	152,5 (8,3)
Other	10 (3,8)	23 (8,6)	100 (9,7)	27 (13,7)	160 (9,1)	91,3 (5,0)
Total	264 (100)	267 (100)	1032 (100)	197 (100)	1760 (100)	1839,3 (100)

*DDD calculations are made only for adults (>15 years).

The most commonly prescribed antibiotics were amoxicillin J01CA04 (33.9% of prescriptions), amoxicillin/clavulanate J01CR02 (18, 7%) and clarithromycin J01FA09 (7.6%).

According to physicians diagnoses the most commonly treated infections, were pharyngitis – 511 patients (29.8%), acute bronchitis - 433 (25.3%), rhinosinusitis – 174 (10.2%) and pneumonia–134 (7.8%). These infections were mainly treated in children (Table 2).

**Table 2:** Infections treated with antibiotics according to patients’ age

**Age group**	**<5**	**5-14**	**15-64**	**> = 65**	**Total**
**Diagnosis**	**N(%)**	**N(%)**	**N(%)**	**N(%)**	**N(%)**
Pharyngitis	103 (39,0)	112 (41,9)	280 (28,4)	16 (8,2)	511 (29,8)
Acute bronchitis	112 (42,4)	65 (24,3)	216 (21,9)	40 (20,4)	433 (25,3)
Rhinosinusitis	27 (10,2)	33 (12,4)	107 (10,9)	7 (3,6)	174 (10,2)
Pneumonia	9 (3,4)	17 (6,4)	83 (8,4)	25 (12,8)	134 (7,8)
Uncomplicated UTI	4 (1,5)	6 (2,2)	79 (8,0)	31 (15,9)	120 (7,0)
Complicated UTI	2 (0,8)	5 (1,9)	40 (4,1)	24 (12,3)	71 (4,1)
Skin and soft tissues infection	2 (0,8)	8 (3,0)	47 (4,8)	13 (6,7)	70 (4,1)
Chronic bronchitis	1 (0,4)	0	36 (3,7)	20 (10,3)	57 (3,3)
Other	4 (1,5)	21 (7,9)	97 (9,8)	19 (9,7)	142 (8,2)
Total	264 (100)	267 (100)	986 (100)	196 (100)	1713 (100)

Pneumonia was primarily treated with macrolides J01FA (clarythromycin, erythromycin and azithromycin) (26,4% of prescriptions for this indication) amoxicillin/clavulanate J01CR02 (25.7%), and amoxicillin J01CA04 (15.7%). None of the patients with pneumonia were prescribed phenoxymetilpenicillin (J01CE02). Antibiotics with poor activity against *Streptococcus pneumonia* (ciprofloxacin J01MA02, cefazolin J01DB04, trimetroprim/sulfometoxazole J01EE01) were prescribed in 5.7% of the pneumonia cases. One hundred twenty eight patients received monotherapy, but six patients – two antibiotics.

Uncomplicated urinary tract infection (120 cases) was mostly treated with fluoroquinolones J01MA (41,7%), oral furazidine J01XE (27.5%), and trimetroprim/sulfomethoxazole (10.8%).

Pharyngitis (511 cases) was mainly treated with amoxicillin (46.3%), amoxicillin/clavulanate (19.1%), trimethroprim/sulfamethoxazole (8.8%). Phenoxymethylpenicillin accounted only for 14 (2%) prescriptions for this infection.

## Discussion

The rapid rise of antimicrobial resistance in ambulatory setting described in the literature has lead to increased interest in understanding how and why outpatient antibiotics are being prescribed. Since there is no computerized medical record system in Latvia, descriptive studies are probably the most efficient way to obtain reliable information.

The study described is the first analysis on antibiotic prescription patterns in Latvia conducted by using an easy-to-understand protocol thus allowing us to obtain information on antibiotic consumption, data on indication for antibiotic use and demographic data on patients treated with antibiotics in ambulatory care settings.

Some type of upper respiratory tract infection was the most common indication for prescribing an antibiotic. This finding is rather similar to what has been found in other studies using different methodology [7,9]. Interestingly diseases treated the most frequently were acute bronchitis, pharyngitis and rhinosinusitis that are usually caused by viruses and in most of the cases are self-limited. We did not have further details on how the physicians made the diagnosis. The authors assume that some patients may have had a prolonged course of disease and some additional risk factors that would require prescribing an antibiotic. Nevertheless, these findings indicated to further training needs on etiology and treatment of the mentioned diseases.

The main group of antibiotics used was broad spectrum penicillins that are considered rather safe ecologically and produce lower antibiotic resistance selection pressure then quinolones, cephalosporins and macrolides. Nevertheless, we observed very little use of phenoxymethylpenicillin that could be an option of choice for the treatment of streptococcal pharyngitis and other respiratory tract infections In addition, rapid antigen detection test is being covered by the healthcare funds. We think that low use of this antibiotic could be associated with cost considerations (it is significantly more expensive than amoxicillin in Latvia) and for several years its availability in pharmacies was quite limited. Future interventions should be directed to promotion and increased availability of this drug that might lead also to cost reductions.

The habit to prescribe more broad-spectrum, newer and more expensive antibiotics combined with alarming increase of antibiotic resistance problems emphasize the need for the implementation of guidelines advocating a restricted use of antimicrobial agents. Antibiotics prescribed for selected indications causes a considerable alarm. Wide use of amoxicillin/clavulanate for treatment of pneumonia, quinolones for uncomplicated urinary tract infection and broad spectrum penicillin for pharyngitis indicates the unnecessary broadening of the antibiotic spectrum and could lead to additional antibiotic resistance selection pressure. Ambulatory use of quinolones for treatment of urinary tract infection has become a common practice and is a cause of considerable alarm due to rapid worldwide spread of highly resistant *Escherichia coli* strains [3,14].

Twenty one different antibiotics were prescribed in children younger than 10 years. We consider suchvariety of medicines used excessive; besides this could indicate the lack of implemented guidance.

Mean antibiotic treatment time was 6.7 days with most of the patients prescribed 7 day treatment course. There are diseases that require prolonged treatment, but recent findings indicate that three-days antibiotic course, if even that is needed, would be sufficient for treatment of most upper respiratory infections that were the main indications for treatment in our study [7,9].

A large proportion of the patients treated with antibiotics were children, which has been a consistent observation in other countries, too [7,9]. They were mostly treated for upper respiratory tract infections that are predominantly caused by viruses in this age. Therefore, despite the low rate of antibiotic use in ambulatory patients in Latvia, which is one of the lowest in Europe, there is still ample opportunity to further reduce antibiotic use by implementing guidelines for management of upper respiratory tract infections.

A one-week point prevalence approach for study of antimicrobial use in ambulatory use has been used in other countries, but other investigators surveyed all patients with infection with a protocol that was rather complicated and time-consuming [7,9]. After having had discussions with Latvian GP representatives the authors of the study decided to simplify the protocol with a view to enable higher compliance. The only denominator used was the number of outpatient consultations during the study period. The other limitation was that the protocol did not allow us to obtain additional demographic information on patients with infection that were not treated with antibiotics. This is assumed to be instrumental in enabling proper statistical analysis on several confounding factors. In future studies involving a smaller number of physicians this aspect shall be considered.

The authors of the study hold the view that the return rate of forms was sufficiently high to answer the proposed study questions and covered close to 500 000 persons of catchment area from the population of Latvia (from different regions). By the same token, those GPs who attended the conference and responded could be more motivated and interested in the subject of antibiotic use and antimicrobial resistance. In addition, active data collection itself may also influence the prescription habits of GPs. Therefore, our findings could be biased towards better quality of prescriptions and the actual situation would reveal more variety and improper treatment. Feedback from the study was provided to all GPs, with a view *inter* to raise awareness of the problem in non- participants.

## Conclusions

Methodology employed provided useful additional information on ambulatory practice of prescribing antibiotics and could be used in further assessment studies. Educational interventions should be focused on treatment of acute pharyngitis and bronchitis in children and unnecessary use of quinolones in adults for uncomplicated urinary tract infection.

## Abbreviations

GP: General practitioner.

## Competing interests

The authors declare that they have no competing interests.

## Authors’ contributions

UD was the study principal investigator, directed the study design/methodology, and drafted the manuscript, ED did the statistical data analysis and editing the manuscript, MA did the data acquisition and preliminary analysis of results, ET and SM has participated in study design and editing the manuscript. All authors read and approved the final manuscript.

## Pre-publication history

The pre-publication history for this paper can be accessed here:

http://www.biomedcentral.com/1471-2296/14/9/prepub
